# DeepOM: single-molecule optical genome mapping via deep learning

**DOI:** 10.1093/bioinformatics/btad137

**Published:** 2023-03-17

**Authors:** Yevgeni Nogin, Tahir Detinis Zur, Sapir Margalit, Ilana Barzilai, Onit Alalouf, Yuval Ebenstein, Yoav Shechtman

**Affiliations:** Russel Berrie Nanotechnology Institute, Technion, Haifa 320003, Israel; Raymond and Beverly Sackler Faculty of Exact Sciences, Center for Nanoscience and Nanotechnology, Tel Aviv University, Tel Aviv 6997801, Israel; Raymond and Beverly Sackler Faculty of Exact Sciences, Center for Nanoscience and Nanotechnology, Tel Aviv University, Tel Aviv 6997801, Israel; Department of Biomedical Engineering, Technion, Haifa 320003, Israel; Department of Biomedical Engineering, Technion, Haifa 320003, Israel; Lorry I. Lokey Center for Life Sciences and Engineering, Technion, Haifa 320003, Israel; Raymond and Beverly Sackler Faculty of Exact Sciences, Center for Nanoscience and Nanotechnology, Tel Aviv University, Tel Aviv 6997801, Israel; Department of Biomedical Engineering, Tel Aviv University, Tel Aviv 6997801, Israel; Russel Berrie Nanotechnology Institute, Technion, Haifa 320003, Israel; Department of Biomedical Engineering, Technion, Haifa 320003, Israel; Lorry I. Lokey Center for Life Sciences and Engineering, Technion, Haifa 320003, Israel

## Abstract

**Motivation:**

Efficient tapping into genomic information from a single microscopic image of an intact DNA molecule is an outstanding challenge and its solution will open new frontiers in molecular diagnostics. Here, a new computational method for optical genome mapping utilizing deep learning is presented, termed DeepOM. Utilization of a convolutional neural network, trained on simulated images of labeled DNA molecules, improves the success rate in the alignment of DNA images to genomic references.

**Results:**

The method is evaluated on acquired images of human DNA molecules stretched in nano-channels. The accuracy of the method is benchmarked against state-of-the-art commercial software Bionano Solve. The results show a significant advantage in alignment success rate for molecules shorter than 50 kb. DeepOM improves the yield, sensitivity, and throughput of optical genome mapping experiments in applications of human genomics and microbiology.

**Availability and implementation:**

The source code for the presented method is publicly available at https://github.com/yevgenin/DeepOM.

## 1 Introduction

Optical genome mapping (OGM) of DNA (Levy-Sakin and Ebenstein 2013; Müller and Westerlund 2017; Gruszka et al. 2021) involves the imaging of fluorescently labeled DNA molecules and their alignment to reference genome sequences. Consequently, the resulting best-matching alignment reports on the exact position of this molecule fragment in one of the organism’s chromosomes. This information enables multiple applications in molecular diagnostics and in genomic research.

Example applications of OGM include species identification (Bouwens et al. 2020; Wand et al. 2019; Grunwald et al. 2015; Müller et al. 2020) for applications such as pathogen identification in clinical samples, as well as genome-wide mapping of effects such as DNA damage (Torchinsky et al. 2019), methylation (Sharim et al. 2019), and structural variations (Ebert et al. 2021). OGM holds several advantages compared to DNA sequencing; for one, it produces extremely long reads of potentially megabase size, which are necessary for mapping large-scale structural and copy number variations in the genome. Additionally, as a single-molecule technique, it holds the potential for extremely high sensitivity, i.e. detection of low quantities of target DNA (Margalit et al. 2021), which is necessary in applications such as cultivation-free pathogen identification (Müller et al. 2020).

Given an image of a DNA molecule labeled at a specific sequence motif, multiple computational approaches have been proposed for its alignment to a reference genome sequence. If the labelling is sparse enough so that individual fluorescent labels can be separated, the positions of the labels are determined using standard localization techniques, such as emitter centroid fitting (Lelek et al. 2021). Then, dynamic programming algorithms (Valouev et al. 2006) are employed to align the label positions to the expected positions of the labeled motif in a reference genome sequence. When the labeled motif is dense in the genome and does not allow for the separation of individual labels, a different approach was used (Bouwens et al. 2020; Wand et al. 2019; Grunwald et al. 2015; Müller et al. 2020), in which the intensity profile along the imaged molecule is aligned by cross-correlation to the theoretical intensity profile expected from the density of the labeled motif in the reference genome.

The accuracy of OGM can be defined as the expected fraction of imaged molecules that are aligned with high confidence to the reference genome. This accuracy is extremely important for applications where the target DNA quantity in the sample is limited, such as cultivation-free pathogen identification (Müller et al. 2020), or where maximal coverage of the genome is required per mapping experiment, such as in rare variant detection (Margalit et al. 2022) or epigenetic mapping (Gabrieli et al. 2018, 2022). The current computational approaches are limited in accuracy since they are unable to extract all the available information from the image of the DNA molecule. Specifically, when emitters are overlapping inside a diffraction-limited spot, classic approaches usually cannot separate them.

In this study, in order to maximize the information extracted from the molecule image, a deep learning approach is presented. Convolutional neural networks (CNNs) were previously shown to become the state-of-the-art for single molecule localization microscopy (SMLM) (Nehme et al. 2018, 2020; Speiser et al. 2021). Here, a similar approach is applied to OGM, and its advantage is demonstrated in images of sparsely labeled DNA molecules stretched in nanochannels. The alignment accuracy of the presented method DeepOM is compared against the commercial Bionano Solve software which localizes sparse emitters, neglecting their diffraction-limited image overlap. In contrast, the localization neural network of DeepOM enables the separation of multiple fluorescent emitters that are within a diffraction-limited spot. Since the probability for wrong alignment of optical maps, as was theoretically shown, depends exponentially on the number of localized labels in the query molecule (Anantharaman and Mishra 2001), the detection of more labels per kilobase of DNA by DeepOM, results in a significantly higher alignment success rate.

## 2 Materials and methods

### 2.1 The DeepOM method

**Figure 1:**
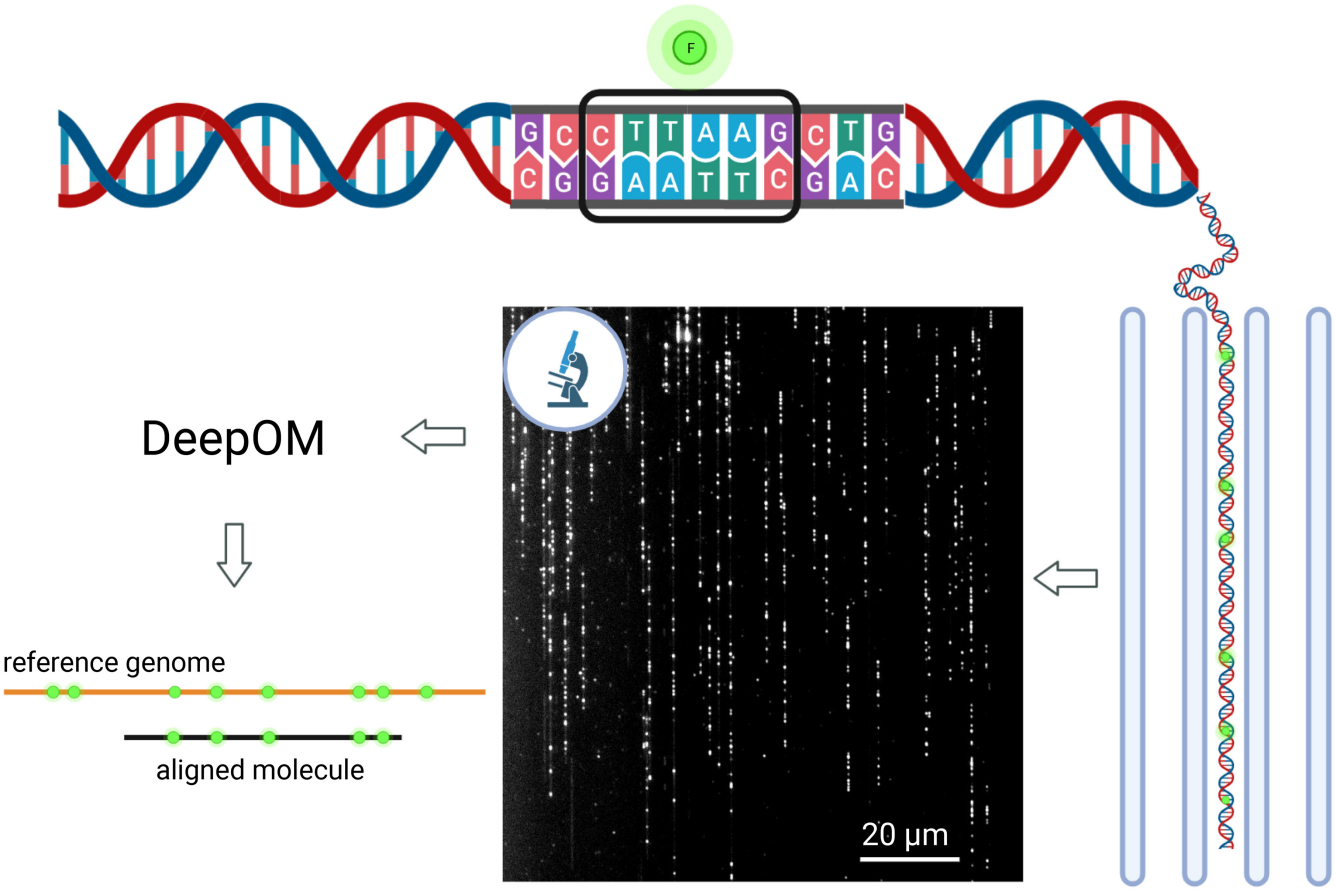
Optical genome mapping using DeepOM. DNA molecules are fluorescently labeled at specific sequence motifs, CTTAAG in this study. Then, they are stretched in nano-channels and imaged in a microscope. The images are analyzed by the DeepOM software, and each molecule is aligned to its top matching position in one of the reference genome sequences

The DeepOM alignment of a DNA molecule to a reference genome sequence starts from query images of molecules fluorescently labeled at specific motifs (Fig. 1). The motif CTTAAG (referred to as DLE-1 by Bionano Genomics) was labeled in this study. In each molecule image, the labels are localized by a localization neural network, resulting in a query map of 1D pixel positions of labels along the length of the molecule. A reference map is the sequence of base-pair positions of the labeled motif in a reference genome sequence. The resulting query map is aligned to the reference map with the dynamic programming alignment algorithm presented below.

### 2.2 Localization neural network

**Figure 2:**
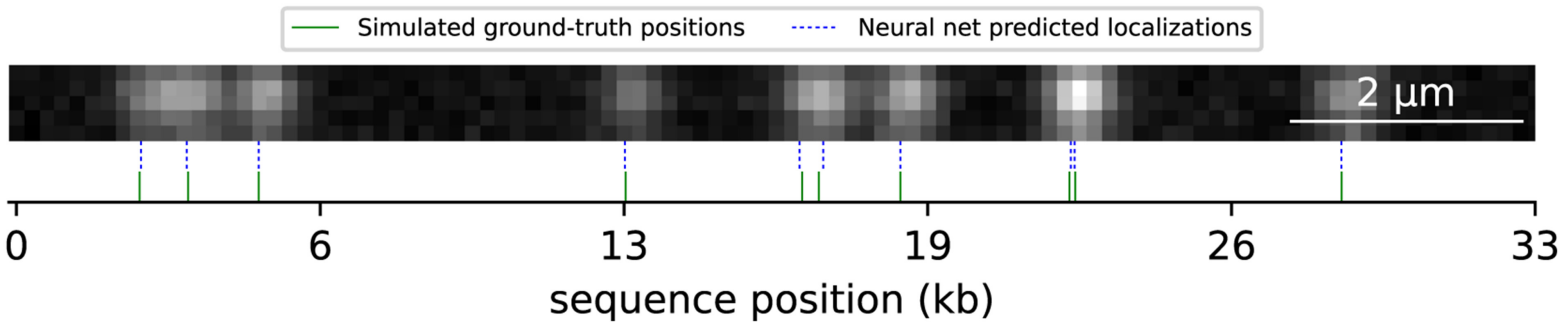
Simulated DNA molecule used for localizer neural net training. Ground-truth label positions and the predicted localizations by the neural net are shown. Random generated emitter positions were confined to a straight line segment, convolved with a 2D Gaussian point-spread function (PSF), and noise was added to the image

A localization neural net model was trained following DeepStorm (Nehme et al. 2018), DeepStorm3D (Nehme et al. 2020), and DECODE (Speiser et al. 2021), where the models are trained in a supervised manner on simulated images from randomly generated ground-truth emitter positions, derived using an optical forward model. Here, a 2D Gaussian point-spread function (PSF) was used for the optical forward model, and emitter positions were confined to a straight line segment (Fig. 2). Following DECODE (Speiser et al. 2021), and DeepStorm (Nehme et al. 2018), a U-Net (Milletari et al. 2016) was used, but with 1D convolutional layers instead of 2D convolutional layers. The input image to the network, which is five pixels wide and 100–1000 pixels long (depending on the length of the molecule), was regarded as a 1D image with five channels (one channel per image row). The last layer of the U-Net was modified to output two 1D vectors, which correspond to two output numbers per 1D pixel: (a) Occupancy probability, i.e. the probability for having an emitter in a pixel and (b) the relative position of the emitter inside the pixel if the pixel contains an emitter. This is valid assuming there is at most one emitter per pixel, which is a good approximation for most datasets of interest, including the one presented here. For the two neural network output numbers defined above, the loss *L* is computed as a sum of two loss terms: the occupancy loss *L*_occ_, and the localization loss *L*_loc_,


(1) $$L(\Omega ,\hat{\Omega },\Lambda ,\hat{\Lambda })=L_{\mathrm{occ}}(\Omega ,\hat{\Omega })+\hat{\Omega }L_{\mathrm{loc}}(\Lambda ,\hat{\Lambda }),$$

where $\Omega_{i}$ is the predicted probability for having an emitter in a pixel *i*; ${\hat{\Omega }}_{i}$ is the ground-truth emitter existence in the pixel, equal to 1 if an emitter is in a pixel and 0 otherwise; $\Lambda_{i}$ is the relative position of an emitter inside the pixel ranging from 0 for the left pixel edge to 1 for the right pixel edge. This position has a meaningful value only in the pixels containing emitters, so the localization loss *L*_loc_ is masked with $\hat{\Omega }$ in the equation; ${\hat{\Lambda }}_{i}$ is the ground-truth relative position of an emitter inside the pixel computed from ground-truth emitter positions, which are known in the simulated data. The loss terms themselves were computed as,


(2) $$L_{\mathrm{occ}}(\Omega ,\hat{\Omega })=L_{\mathrm{dice}}(\Omega ,\hat{\Omega })+\sum_{i}L_{\mathrm{bce}}(\Omega_{i},{\hat{\Omega }}_{i}),$$

(3) $$L_{\mathrm{loc}}(\Lambda ,\hat{\Lambda })=\sum_{i}L_{\mathrm{bce}}(\Lambda_{i},{\hat{\Lambda }}_{i}),$$

where the *i*-summation is over the 1D pixel indices along the length of the molecule image, and with Dice-Loss (Sudre et al. 2017) $L_{\mathrm{dice}}$ and Binary-Cross-Entropy $L_{\mathrm{bce}}$ defined as,


(4) $$L_{\mathrm{dice}}(\Omega ,\hat{\Omega })=1-\frac{10^{-5}+2\sum_{i}\Omega_{i}{\hat{\Omega }}_{i}}{10^{-5}+\sum_{i}\Omega_{i}+\sum_{i}{\hat{\Omega }}_{i}},$$

(5) $$L_{\mathrm{bce}}(x,y)=-x\;\text{log}(y)-(1-x)\;\text{log}(1-y).$$

In each gradient descent training step, the model was presented with a batch of randomly generated DNA molecule images, and the loss was computed as described above using the ground truth positions of emitters in the molecule (Fig. 2). Training was done for 10 000 steps with a 256 batch size. More training details are given in Supplementary Fig. S1 in Supplementary Appendix S1, and network architecture is described in Supplementary Appendix S2.

### 2.3 Training dataset

The training dataset was generated by simulating DNA molecule images with randomly generated emitter positions, representing randomly generated genome sequences as shown in Fig. 2. The following average parameter values were used for the generation of the images: pixel size of 335 basepairs, average emitter density of 1 emitter per 4096 basepairs (each basepair has a 1/4096 probability of containing an emitter), Gaussian point spread function (PSF) with a standard deviation of 1.5 pixels, signal-to-noise ratio (SNR) of 2.33. In contrast to the training dataset, the test dataset used in this study consisted of experimental images of DNA molecules, originating from the real human genome. In the same manner as in DECODE (Speiser et al. 2021), the training images were continuously generated and each image is used only once as a training target. For this reason, the training dataset is infinite and the model cannot overfit to the training images. Thus, no separate validation dataset is necessary (Speiser et al. 2021) (the new images serve as validation), and the test set consisting of experimental DNA molecules, was used to evaluate the model.

### 2.4 Alignment algorithm

The algorithm by Valouev et al. (2006) was implemented to align the localized labels in a DNA molecule to the reference genome. An alignment of a DNA query molecule and a reference genome sequence is a set of labeled position pairs from the query and reference. The implementation of the algorithm computes the following dynamic programming recurrence equation for the alignment score matrix $S_{i,j}$,


(6) $$S_{i,j}=1+\underset{\begin{array}{c} i-\delta \leq g<i\\ j-\delta \leq h<j \end{array}}{\max}\left\{\begin{array}{c} S_{g,h}+\\ -\alpha^{-1}\left|\left|r_{i}-r_{g}|-|q_{j}-q_{h}\right|\right|+\\ -\beta^{-1}\left|i-g-1+j-h-1\right|+ \end{array}\right\}.$$

Then, the alignment is traversed back starting from the maximal cell value in the score matrix. *r_i_* is the reference positions vector indexed by the integers *i* or *g*, and *q_j_* is the query positions vector indexed by the integers *j* or *h*. The query vector q=sx is obtained by converting the pixel value *x* of localized emitters to basepairs through a conversion scale factor $s=335\frac{bp}{\mathrm{pixel}}$. *δ* = 5 is the allowed margin for missing labels in query or reference, *α* = 500 is the penalty factor for localization error, *β* = 10 is the penalty factor for a missing label in the alignment. $S_{i,j}$ is the score of the top-scoring alignment of *r_i_* and *q_j_* ending in indices *i*, *j*.

## 3 Sample preparation and imaging

### 3.1 Cell culture

U2OS (human OS) cell line was cultured in Dulbecco’s Modified Eagle medium, supplemented with 10% fetal bovine serum (Gibco, Amarillo, TX), 2 mM l-glutamine, and 1% penicillin-streptomycin (10 000 U/ml; Gibco) and incubated at 37°C with 5% CO_2_.

### 3.2 DNA extraction

DNA was extracted from 10^6^ cells using the Bionano Prep Cell Culture DNA Isolation Protocol according to manufacturer’s instructions.

### 3.3 DNA labeling

One microgram of DNA was directly labeled and stained using DLS labeling kit (Bionano Genomics) composed of a single enzymatic labeling reaction with DLE-1 enzyme followed by DNA staining with a fluorescent marker. One microgram of DNA was mixed with 6 µl of 5× DLE-buffer, 2 µl of 20× DL-Green, and 2 µl of DLE-1 enzyme (Bionano Genomics) in a total reaction volume of 30 µl and incubated for 2 h at 37°C.

### 3.4 DNA imaging

DNA image data were generated on the Saphyr instrument (Bionano Genomics) with Saphyr chips (G1.2). The chip was loaded as recommended by Bionano Genomics.

## 4 Results and discussion

**Figure 3:**
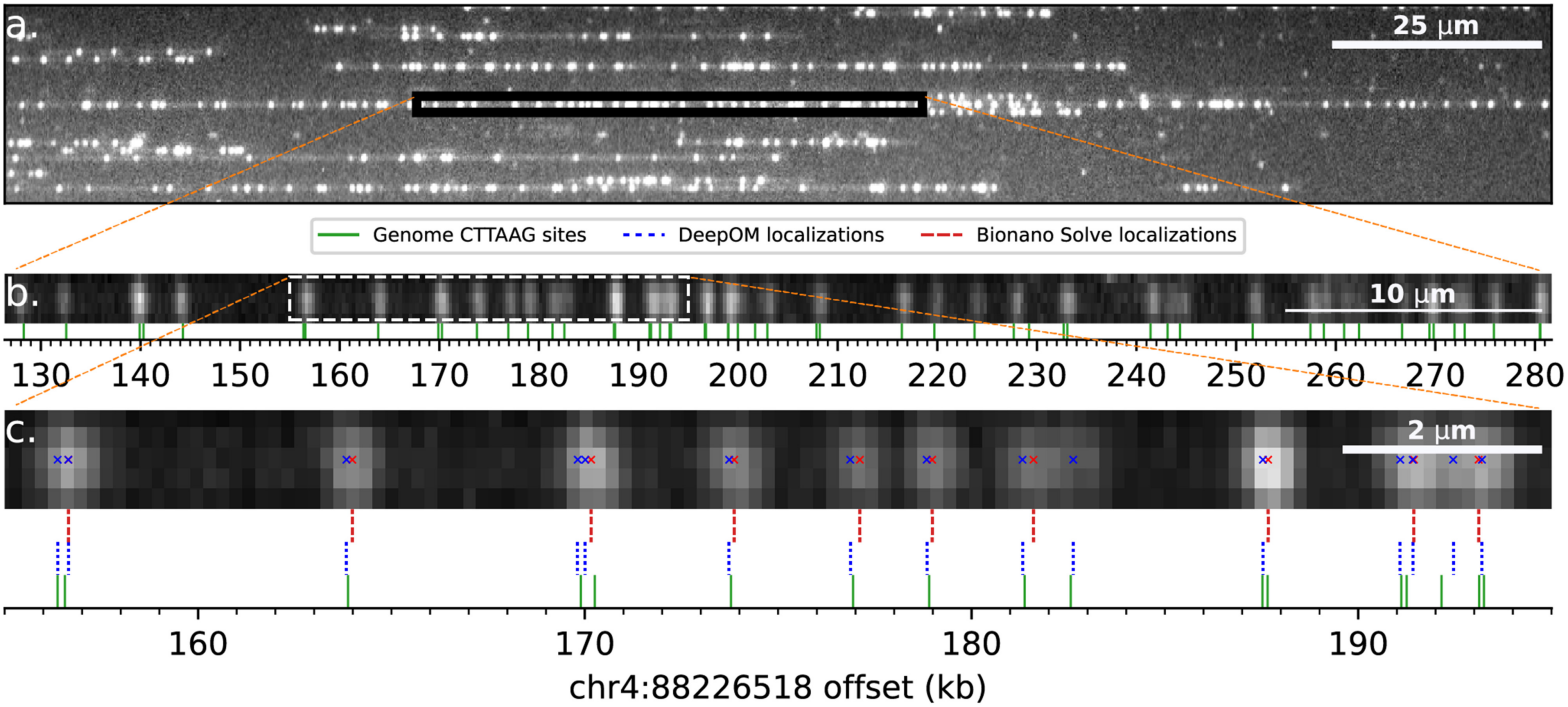
Experimentally imaged DNA molecules and their alignment to the human genome. (a) Zoomed-in field-of-view of an image captured in the Bionano Saphyr system. DNA molecules are stretched here in nano-channels of the Bionano Chip. (b) Zoomed-in view to a 500 kb molecule from the field-of-view image (a). This molecule is used as a ground-truth for alignment of its cropped sub-fragments. Cropped sub-fragment (white dashed rectangle), zoomed-in in c. The reference genome labeled motif (CTTAAG) sites are shown, in relative offset to human genome coordinates shown on the x-axis. The alignment of the molecule to the reference was done both by Bionano Solve and DeepOM, and the resulting genome coordinates were practically identical. (c) An example cropped fragment used for the alignment success rate comparison. Shown are Bionano Solve localizations, DeepOM localizations. The reference sites genome coordinates of the parent molecule are used as a ground-truth for the evaluation of the success of this fragment’s alignment. The advantage of DeepOM is manifested here, where pairs of tightly spaced labeled motifs are separated by the neural net, while the classical localization approach detects only one label at the diffraction limited spot. This in turn, leads to more confident and accurate alignments with higher success rates

The accuracy of DeepOM’s alignments was evaluated on images (Fig. 3) produced from the Bionano Genomics Saphyr system described in Section 2. The reference genome used for alignments is the reference human genome GRCh38 (Nurk et al. 2022). The ground truth for alignments was generated as follows. All molecules longer than 450 kb were taken from the imaged data, and aligned to the reference genome with the Bionano Solve software. Out of those, the top 512 molecules were chosen by their Bionano alignment confidence score (see Bionano documentation https://bionanogenomics.com/support-page/data-analysis-documentation/). Each chosen long molecule image was digitally cropped (Arielly and Ebenstein 2018) into random short fragments (Fig. 3). Since each cropped fragment’s position is known within the parent molecule, its aligned position can be regarded as a ground truth for the purpose of the alignment accuracy evaluation. Each cropped fragment image is fed into the DeepOM pipeline and aligned to the reference genome, then if the alignment matches the ground truth it is counted as correct.

**Figure 4:**
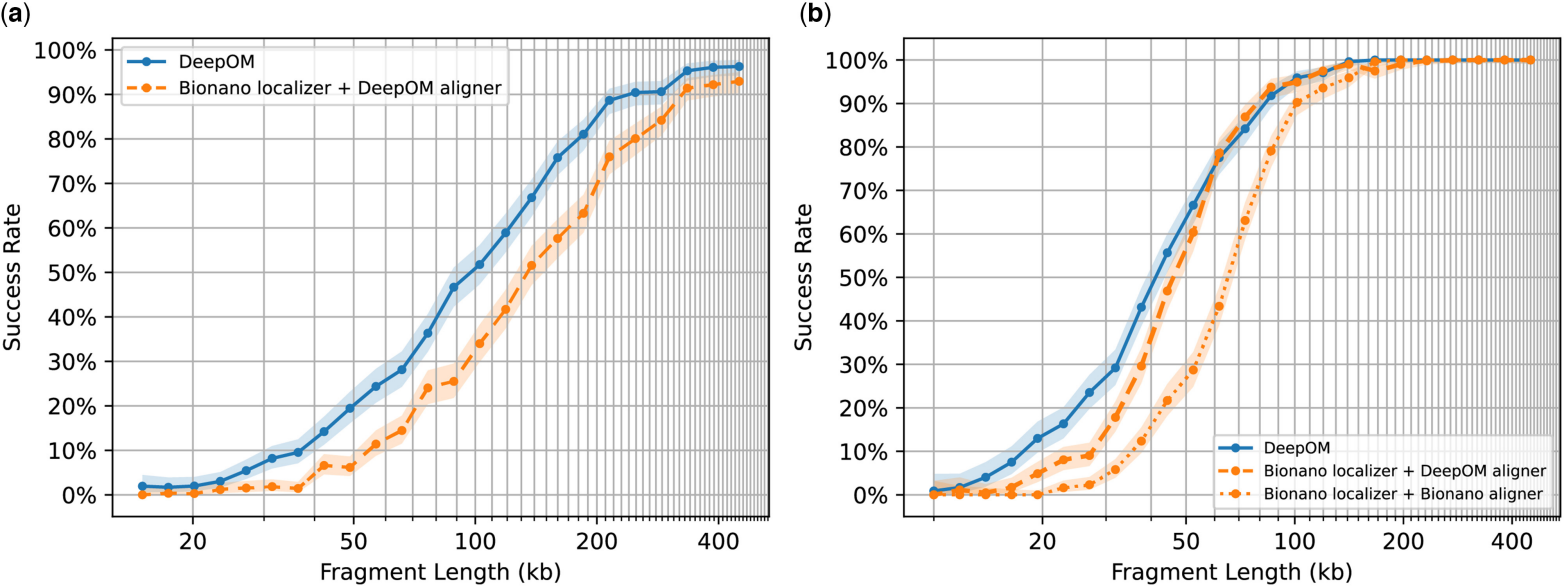
Accuracy evaluation of DeepOM versus Bionano Solve. The alignment success rate is shown for DeepOM and Bionano Solve versus fragment length. The success rate for a fragment length, is defined as the fraction of correct alignments for this length. Each success rate point was computed from 512 random cropped fragments with a ground-truth alignment from long high-confidence aligned molecules (Fig. 3). Each cropped fragment was aligned to the reference genome and the success rate is the fraction of correct alignments, 95% confidence bounds are shown, computed with the Clopper–Pearson interval Beta Distribution (Clopper and Pearson, 1934). In (a), DeepOM is compared against the localizations produced by Bionano Solve, which are aligned to the reference genome, with the DeepOM aligner. While in (b) comparison is also against the whole Bionano Solve pipeline including the Bionano localizer and Bionano aligner (orange dotted line)

To make the comparison to the Bionano Solve software, cropping of the long molecules was done digitally by manipulation of the Bionano BNX output files (see Bionano documentation) produced from the imaging experiment. The BNX file contains a localization list for the emitters in each molecule, and the molecules’ coordinates in the captured field-of-view image. In order to generate the cropped fragments in the BNX file, labels were deleted from the localization list according to the cropped fragments (Fig. 3). First, we demonstrate the effectiveness of dense localization by the neural net, compared to standard, sparse localization, which discards closely spaced emitters. To do so, the localization list for each cropped molecule was aligned to the reference using the DeepOM alignment algorithm (Section 2), and the success rate is shown in Fig. 4a, presenting the comparison of the DeepOM localizer and the Bionano localizer both using the same alignment algorithm of DeepOM.

Next, the whole DeepOM pipeline was compared versus the full Bionano localizer and aligner pipeline, on a subset of molecules. To run the Bionano pipeline, a new BNX input file was generated containing random crops from the chosen molecules. Then, the file was fed as input to the Bionano Solve aligner. The success rate comparison of the full pipelines is shown in Fig. 4b.

In both comparison methods, the results in Fig. 4 show more than a 2-fold improvement factor in the success rate for fragments shorter than 50 kb. Notably, optimizing the alignment algorithm, together with the localization neural net, further improves the alignment success rate, as can be seen when comparing the DeepOM aligner to the Bionano aligner, when both using the same localizer (Fig. 4b). Additional evaluations versus simulated data, versus other localization methods, and comparison of run-times are in Supplementary Appendices S3 and S4.

## 5 Conclusions

In this study, an improved computational method for optical genome mapping was presented. A CNN was employed to significantly improve the success rate of alignments, as compared to a state-of-the-art non-overlapping approach. The accuracy of the presented method, DeepOM, was compared against the state-of-the-art commercial Bionano Solve on human cell-line DNA data acquired with the Bionano Saphyr system. The advantage of the presented method is most dominant for DNA fragments in the range 50–150 kb, where it yields up to twofold more successful alignments (Fig. 4b). This is especially significant given that the Bionano Genomics pipeline recommends filtering out molecules shorter than 150 kb in order to provide a high mapping rate. In contrast, DeepOM allows exploiting the information from these shorter molecules. DeepOM enables higher genome coverage from a given sample, enhancing the ability to detect low-frequency structural variations. The DeepOM method can also be potentially applicable to images of DNA molecules stretched on a free surface and not in nanochannels, provided the appropriate pre-processing and segmentation algorithms for the images. In conclusion, the presented method may be utilized in molecular diagnostic applications such as epigenetic profiling, and pathogen species identification, where it can significantly increase the fraction of identified molecules, enabling higher diagnostic sensitivity.

## Supplementary Material

btad137_Supplementary_Data

## Data Availability

The data underlying this article will be shared upon reasonable request to the corresponding author.

## Code availability

The code to reproduce the figures in this article is available at https://github.com/yevgenin/DeepOM.
