# Supplementary material for: DeepOM: single-molecule optical genome mapping via deep learning
Source: Bioinformatics. 2023 Mar 17;39(3):btad137. doi: 10.1093/bioinformatics/btad137 (PMC10049785; doi:10.1093/bioinformatics/btad137)
Supplement: btad137_Supplementary_Data [file btad137_supplementary_data.pdf]

S1 Training details

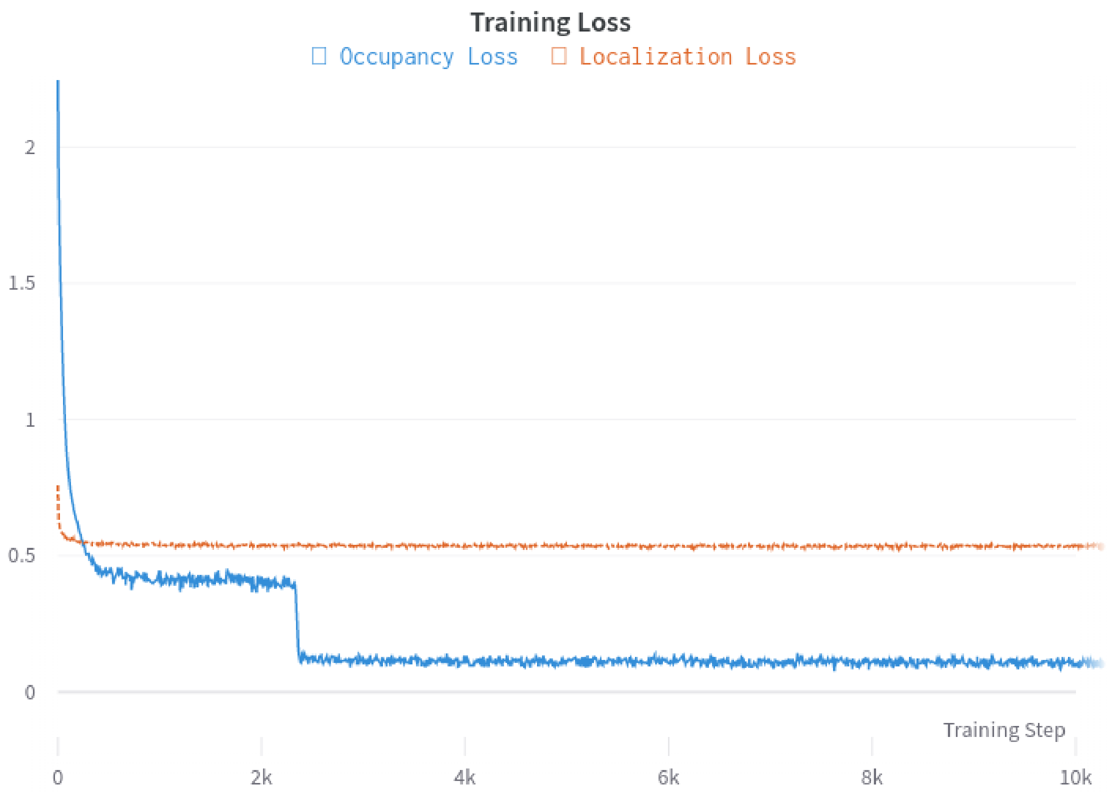

Figure S1: **Training the localization Neural Network model**. The Occupancy $\mathcal{L}_{occ}$ and the Localization losses $\mathcal{L}_{loc}$ described in the Methods section (eq. 2 and 3) are shown for the training of the DeepOM localization neural net model. The model was trained for 10000 training steps with each step having a batch of 256 generated DNA molecule images, giving a total of $256 \times 10000 = 2560000$ simulated DNA molecule images used for training. The training stopping condition was based on the loss terms described in the Methods section achieving a plateau. The training was performed on a single NVIDIA RTX 3090 GPU.

S2 Network architecture

The network architecture implementation that was used in the presented DeepOM method is the

```
monai.networks.nets.BasicUNet
```

from the MONAI library [mon, 2022]. The parameters used were:

```
BasicUNet(
    spatial_dims=1,
    features=[32, 32, 64, 128, 256, 32],
    in_channels=5,
    out_channels=4,
    upsample="pixelshuffle",
    )
```

The above code results in a 1-D U-NET [Falk et al., 2019] architecture with convolutional layers described in Table S1, max-pooling for downscaling, pixelshuffle [Shi et al., 2016] for upscaling, leaky ReLU activation functions, and instance normalization between all layers.

Layer	Input Channels	Output Channels
Input Conv	5	32
Conv 1	32	32
Conv 2	32	32
Conv 3	32	64
Conv 4	64	64
Conv 5	64	128
Conv 6	128	128
Conv 7	128	256
Conv 8	256	256
Conv 9	256	128
Conv 10	128	128
Conv 11	128	64
Conv 12	64	64
Conv 13	64	32
Conv 14	32	32
Conv 15	32	32
Conv 16	32	32
Output Conv	32	4

Table S1: **Convolutional Layers in the 1-D U-NET architecture used in DeepOM.** All layers have kernel size of 3 and stride of 1.

S3 Comparison vs. FALCON and on simulated vs. experimental data

Comparison vs. FALCON Many different Single Molecule Localization (SMLM) algorithms exist, and one of the top performing in the 2D high-spot-density case is FALCON [Min et al., 2014] (as was reviewed and compared in [Sage et al., 2019]). In Figure S2 the accuracy of the DeepOM method is compared vs. FALCON, where DeepOM achieves significantly better results in terms of alignment success rate. The advantage is even more significant for experimental data, meaning that a Deep Learning based method utilized in DeepOM allows for better generalization to the uknown physical model of the experimental images. The run-times of the methods are compared in Table S2.

The parameters used for running the FALCON matlab based software code were:

```
numFrame = 1;
dummyFrame = 0;
Gsigma1 = 1.5;
Gsigma2 = 2;
Gsigma_ratio = 0.8;
EM = 1;
speed = 'normal';
ADU = 1;
debug = 0;
baseline = 5000;
[Results, Avg_img] = FALCON_CPU_rel2(filename, numFrame, dummyFrame, ADU,
    baseline, EM, Gsigma1, Gsigma2, Gsigma_ratio, speed, debug);
```

With the following explanation of the parameters by the authors of FALCON:

```
%% Input:  filename          : file name of raw camera images, ex) xxx.tif
%%         numFrame          : number of frames to be reconstructed
%%         dummyFrame        : number of frames to be discarded first
%%         ADU               : photons per camera unit (important)
%%         baseline          : baseline of camera, ex) 100
%%         pixel_size        : raw camera pixel size in nm
%%         EM                : ON = 1 OFF = 0
%%         Gsigma1 & 2       : widths of two Gaussian fuctions
%%         Gsimga_ratio      : ratio for two Gaussian functions for PSF:
        PSF = Gsimga_ratio*F_sigma1 + (1-Gsimga_ratio)*F_sigma2
%%         Speed             : option for reconstruction speed
%%         debug             : debug mode
```

The parameters above were the default ones taken from the software demo code, except for the baseline parameter, which was adjusted to the background noise level in both the simulated and experimental images. The Gaussian Point Spread Function (PSF) size (sigma) of 1.5 pixels is similar to the value used for the simulated data generated (Figure 2) for training the DeepOM localization model.

Comparison vs. simulated Data The accuracy of the DeepOM method and FALCON localizer is compared vs. simulated data in Figure S2. Simulated data was generated in the same way as described in

the Methods section Figure 2, with the exception that the genome sequences underlying the generated images were fragments from the real human genome, and the emitter spots in the images correspond to positions of the labeled sequences in the reference human genome (see Figure 3). In this way, the evaluation is done on images never seen by the model during training, as the model was trained only on simulated images from synthetic random genomes. As expected, the accuracy of the DeepOM method is better on simulated images than on experimental images, as the training data was generated with the same physical image generation model (point-spread-function, image noise distribution) as was used for the simulated images used for the performance evaluation. In contrast, the exact physical image generation process (point-spread-function, image noise distribution) of the experimental images is not known precisely and will inevitably differ somewhat from the model assumed during training.

	**Run time per molecule image**
FALCON Localizer [Min et al., 2014]	5.65 s ± 47.2 ms
**DeepOM Localizer**	7.77 ms ± 670 µs (**CPU**)<br>6.38 ms ± 810 µs (**GPU**)

Table S2: **Run time comparison for DeepOM localizer vs. FALCON localizer** Average and standard deviation of run times computed over 10 repeated runs are shown. It is evident that the DeepOM localizer is significantly faster than the FALCON localizer. Even when evaluated on CPU without the GPU. This is due to the fact that FALCON is based on an iterative reconstruction algorithm, while DeepOM is based on a single forward pass of the neural network.

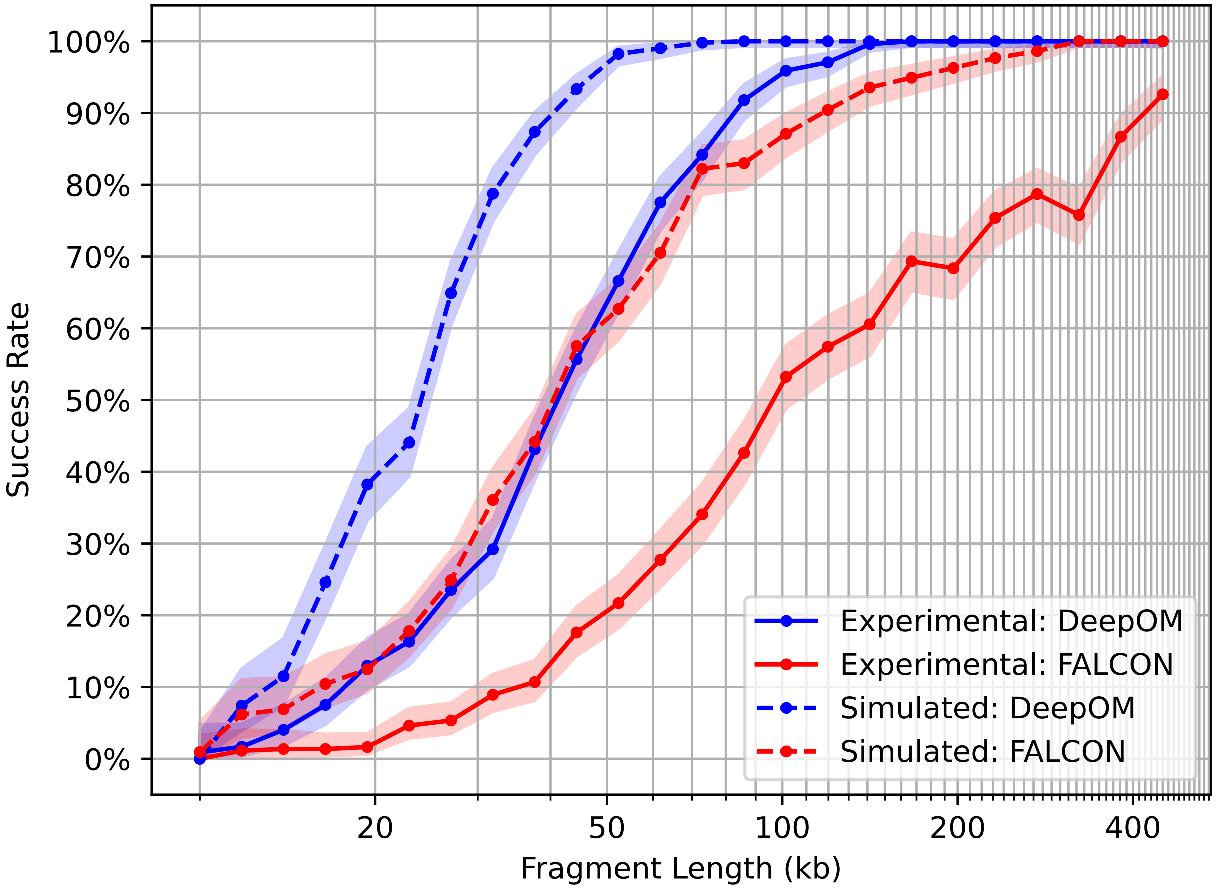

Figure S2: **Comparison of DeepOM and FALCON** [Min et al., 2014] **on experimental and simulated datasets**. Experimental data was the same as used in the comparison vs. the Bionano Software in Figure 4. For both simualted and experimental data, for each fragment length, 512 crops were made from the original selected molecules in the same way as described in Figure 4. In simulated images, the experimental image was replaced by a simulated version generated as described in the Methods section, Figure 2. 95% confidence bounds are shown, computed with the Clopper-Pearson interval Beta Distribution [Clopper and Pearson, 1934]

S4 Run times comparison of DeepOM vs. Bionano software

The Bionano RefAligner was run on experimental data in the form of BNX files produced during the operation of the Bionano Saphyr instrument. The full DeepOM pipeline was run on the same molecule images, pointed to in the BNX files. 100 crops (as was described in Figure 3) of 100kb size (total of 10 megabase) were used for the evaluation of run-time (to amortize the startup time of the Bionano RefAligner software). Run time comparison is in Table S3.

	**Run time per 100 molecule images (10 megabase total)**	**Expected run time per gigabase of material**
Bionano RefAligner	36.9 s ± 864 ms	1.02 hour
**DeepOM (full pipeline)**	35.7 s ± 1.02 s	0.99 hour

Table S3: **Run time comparison of the full pipelines (localizer + aligner) for DeepOM vs. Bionano** Average and standard deviation of run times computed over 10 repeated runs are shown. The expected run time for a gigabase (run-time for 10 megabase multiplied by 100) is shown in the right column. The evaluation was done on a machine with an Intel i9-11900 CPU, 128GB RAM, NVIDIA RTX 3090 GPU. It is important to note that the Bionano localizer runs during the operation of the Saphyr instrument and is not available as an independent software package. Therefore, the run time for the Bionano localizer is not included in the Bionano RefAligner run time. So, the actual run time for the Bionano pipeline is higher than stated here.

References

MONAI: Medical Open Network for AI, Dec. 2022.

C. J. Clopper and E. S. Pearson. The use of confidence or fiducial limits illustrated in the case of the binomial. *Biometrika*, 26(4):404–413, 1934.

T. Falk, D. Mai, R. Bensch, Ö. Çiçek, A. Abdulkadir, Y. Marrakchi, A. Böhm, J. Deubner, Z. Jäckel, K. Seiwald, et al. U-net: deep learning for cell counting, detection, and morphometry. *Nature methods*, 16(1):67–70, 2019.

J. Min, C. Vonesch, H. Kirshner, L. Carlini, N. Olivier, S. Holden, S. Manley, J. C. Ye, and M. Unser. Falcon: fast and unbiased reconstruction of high-density super-resolution microscopy data. *Scientific reports*, 4(1): 4577, 2014.

D. Sage, T.-A. Pham, H. Babcock, T. Lukes, T. Pengo, J. Chao, R. Velmurugan, A. Herbert, A. Agrawal, S. Colabrese, et al. Super-resolution fight club: assessment of 2d and 3d single-molecule localization microscopy software. *Nature methods*, 16(5):387–395, 2019.

W. Shi, J. Caballero, F. Huszár, J. Totz, A. P. Aitken, R. Bishop, D. Rueckert, and Z. Wang. Real-time single image and video super-resolution using an efficient sub-pixel convolutional neural network. In *Proceedings of the IEEE conference on computer vision and pattern recognition*, pages 1874–1883, 2016.
